# Prevention of relapse in drug sensitive pulmonary tuberculosis patients with and without vitamin D3 supplementation: A double blinded randomized control clinical trial

**DOI:** 10.1371/journal.pone.0272682

**Published:** 2023-03-30

**Authors:** Sanjeev Sinha, Himanshu Thukral, Imtiyaz Shareef, Devashish Desai, Binit Kumar Singh, Bimal Kumar Das, Sahajal Dhooria, Rohit Sarin, Rupak Singla, Saroj Kumari Meena, Ravindra Mohan Pandey, Shivam Pandey, Sunil Sethi, Ashumeet Kajal, Rakesh Yadav, Ashutosh Nath Aggarwal, Sanjay Bhadada, Digambar Behera

**Affiliations:** 1 Department of Medicine, All India Institute of Medical Sciences, Ansari Nagar, New Delhi, India; 2 Deparment of Microbiology, All India Institute of Medical Sciences, Ansari Nagar, New Delhi, India; 3 Department of Pulmonary Medicine, Post Graduate Institute of Medical Education and Research, Chandigarh, India; 4 Department of Respiratory Medicine, National Institute of Tuberculosis and Respiratory Diseases, New Delhi, India; 5 Department of Biostatistics, All India Institute of Medical Sciences, Ansari Nagar, New Delhi, India; 6 Department of Medical Microbiology, Post Graduate Institute of Medical Education and Research, Chandigarh, India; Rutgers Biomedical and Health Sciences, UNITED STATES

## Abstract

**Background:**

The immunomodulatory effects of vitamin D are widely recognized and a few studies have been conducted to determine its utility in the treatment of tuberculosis, with mixed results. This study was conducted to see if vitamin D supplementation in patients with active pulmonary tuberculosis (PTB) in the Indian population contributed to sputum smear and culture conversion as well as the prevention of relapse.

**Methods:**

This randomized double-blind placebo-controlled trial was conducted in three sites in India. HIV negative participants aged 15–60 years with sputum smear positive PTB were recruited according to the Revised National Tuberculosis Control Program guidelines and were randomly assigned (1:1) to receive standard anti-tubercular treatment (ATT) with either supplemental dose of oral vitamin D3 (60,000 IU/sachet weekly for first two months, fortnightly for next four months followed by monthly for the next 18 months) or placebo with same schedule. The primary outcome was relapse of PTB and secondary outcomes were time to conversion of sputum smear and sputum culture.

**Results:**

A total of 846 participants were enrolled between February 1, 2017 to February 27, 2021, and randomly assigned to receive either 60,000 IU vitamin D3 (n = 424) or placebo (n = 422) along with standard ATT. Among the 697 who were cured of PTB, relapse occurred in 14 participants from the vitamin D group and 19 participants from the placebo group (hazard risk ratio 0.68, 95%CI 0.34 to 1.37, log rank p value 0.29). Similarly, no statistically significant difference was seen in time to sputum smear and sputum culture conversion between both groups. Five patients died each in vitamin D and placebo groups, but none of the deaths were attributable to the study intervention. Serum levels of vitamin D were significantly raised in the vitamin D group as compared to the placebo group, with other blood parameters not showing any significant difference between groups.

**Conclusions:**

The study reveals that vitamin D supplementation does not seem to have any beneficial effect in the treatment of PTB in terms to the prevention of relapse and time to sputum smear and culture conversion.

**Trial registration:**

CTRI/2021/02/030977 (ICMR, Clinical trial registry-India).

## Background

Tuberculosis (TB) is a debilitating contagious disease and a major global public health problem [1] A total of 1.5 million people died from TB in 2020 (including 214,000 people with HIV). Worldwide, TB is the 13th leading cause of death and the second leading infectious killer after COVID-19 (above HIV/AIDS). Eight countries account for two thirds of worldwide deaths, with India leading the count, followed by China, Indonesia, the Philippines, Pakistan, Nigeria, Bangladesh and South Africa [2].

The high morbidity and mortality contributed by PTB necessitates supplemental management strategies in addition to the existing anti-microbial treatment. The concept of Host-directed therapy (HDT) has gained momentum in recent years, which aims at modifying the host cell functions to counteract the damage and complications caused by the pathogen. A number of such adjunctive strategies has been studied, which includes Vitamin D, Pentoxifylline, Prednisolone, GM-CSF, Recombinant human interleukin, N-acetylcysteine, Immunoxel etc., with generally favorable results [3, 4].

Vitamin D, also called cholecalciferol, is a micronutrient which is recognized primarily for its role in bone mineralization. It has also been under scrutiny lately with respect to its immuno-modulatory properties. In recent years, extensive research has been done to look for other functions of vitamin D and its related metabolites. Many epidemiological studies have shown inverse relation in the levels of vitamin D and incidence of various infectious and chronic diseases [5]. The major circulating metabolite of vitamin D is 1,25-hydroxyvitamin D (1,25[OH]D), which supports innate antimicrobial immune responses, thus suggesting a potential mechanism by which adjunctive vitamin D might enhance response to anti-tuberculosis therapy (ATT) [6].

Consequently, in a country with a high TB burden such as India, there is a compelling need to investigate the potential benefits of vitamin D supplementation along with ATT in participants with active pulmonary TB (PTB), particularly with regards to preventing relapse after successful treatment completion and cure as well as achieving early sputum and culture conversion. If so, it can be helpful in further optimizing the management of PTB. Hitherto few studies have been conducted to investigate the effect of vitamin D in the management of PTB, albeit with small sample sizes, limited dosage of vitamin D and with analysis of limited parameters in India [7]. This randomized control trial was done to assess the effect of vitamin D in patient with PTB on the time to sputum smear and culture conversion as well as the risk of relapse.

## Methods

### Study design and study area

We conducted a randomized double-blind placebo-controlled trial at three tertiary care hospitals in India (All India Institute of Medical Sciences, Delhi; National Institute of Tuberculosis and Respiratory Diseases, Delhi and Post Graduate Institute of Medical Education and Research, Chandigarh).

### Study population

Patients were recruited from DOTS (Directly Observed Treatment, Short Course) centers in agreement with Revised National Tuberculosis Control Program (RNTCP) guidelines [8]. In accordance with the guidelines, an individual was designated as a Pulmonary TB suspect if he/she was having a cough of 2 weeks or more duration, with or without other symptoms, or was a contact of smear positive TB patient and having cough of any duration. Two sputum examination was performed in such individuals, with one of them being a morning sputum specimen. New sputum smear positive participants in the age group of 15–60 years and willing to participate were enrolled for the study. Participants having any major surgical or medical illness or requiring hospitalization for the same or having chronic kidney disease, having MDR-TB as per the results of First Line LPA, Human Immunodeficiency Virus (HIV) infection, BMI < 15kg/m2, diabetes mellitus, chronic alcoholism, pregnant and lactating women, or receiving any kind of vitamin D / calcium supplementation or having any disorder of bone mineral homeostasis, and those refusing consent were excluded from the study.

Participants were diagnosed as a case of PTB by sputum smear examination for acid fast bacilli (AFB), for which 2 sputum samples were collected- one early morning and the second on the spot. The sputum was treated with N-acetyl-L-cysteine/NaOH and the ensuing sediment was stained by Ziehl Neelsen method [9]. The slides were examined under oil immersion objective, and the number of AFB observed were graded from scanty to 3+ as per the recommendations of WHO. Sputum cultures were done using the Mycobacteria Growth Indicator Tube (MGIT-960) non radiometric automated isolation system (Becton Dickinson), and drug resistance was determined using the line probe assay (LPA) method [10]. To ensure eligibility of participants, pre-randomization investigations for hepatic and renal assessment, serology for HIV, Hepatitis B and Hepatitis C as well as fasting blood glucose were done. Other investigations like chest radiograph, electrocardiogram, complete hemogram and urine microscopy were also done.

The primary outcome was rate of relapse of PTB in both arms assessed up to 24 months of follow up. Secondary outcomes included time to sputum smear and culture conversion in months.

### Randomization

Participants were randomly assigned (1:1) into two groups by using block randomization with a block size of 4 using a computer program. Packets containing the two interventions were prepared in accordance with the randomization numbers and were arranged serially, and then distributed consecutively to participants according to their entry into the trial. Both the groups received standard DOTS ATT as per RNTCP guidelines, according to which they received Isoniazid, Rifampicin, Pyrazinamide and Ethambutol thrice a week on alternate days for 2 months- designated as the Intensive phase, which was followed by the Continuation phase where they received Isoniazid, Rifampicin thrice a week on alternate days for 4 months). However, after 1 year of study initiation, patients were provided with daily Isoniazid, Rifampicin, Pyrazinamide and Ethambutol for 2 months during the Intensive Phase followed by daily Rifampicin, Isoniazid and Ethambutol for 4 months during the Continuation Phase, in accordance with the changes proposed by the RNTCP (8). Those with persistent sputum AFB smear positive at two months of therapy were provided an extra month of intensive therapy The intervention group received supplemental dose of oral vitamin D3 (60,000 IU/sachet every week for first two months, then every fortnight for next four months and then every month for next 18 months), and the control received only placebo with an identical schedule. The vitamin D and placebo sachets were identical in terms of their taste and physical appearance.

### Procedures

Sputum smear for AFB was taken every fortnight during the intensive phase (two months), every month during the continuation phase (four months) and once every six months thereafter till the 24^th^ month of follow up. Samples for MGIT culture were taken every fortnight for the initial 2 months and every month thereafter till 6 months of follow up. Sputum samples were taken twice, with one of them being a morning sample for each visit. Sputum induction was performed for those patients who were unable to produce quality sputum specimens, especially for those who had resolution of cough early in the course of treatment. Blood and urine investigations were conducted every fortnight till two months, then every month till six months and then every six months till the 24^th^ month follow up. Chest radiograph was done at baseline, at the end of the intensive phase, end of treatment and every 6 months thereafter. Ultrasound abdomen done at baseline, end of treatment and at 24 months. Disease severity was graded on chest radiographs as minimal, moderately advanced, and far advanced according to the American Thoracic Society recommendations [11].

### Definitions

Individuals were followed up regularly as per the plan mentioned before, to detect for sputum smear conversion (From AFB positive smear during recruitment to AFB negative during the treatment phase with ATT), Sputum Culture Conversion (From MGIT culture positive at recruitment to MGIT culture negative during the treatment phase with ATT). Treatment was considered complete if the subject completed both the intensive and continuation phase without any clinical deterioration and microbiological evidence of disease. Subject was declared cured after treatment completion when both sputum smear and Sputum Culture samples were negative for M tuberculosis. Relapse was defined in a patient who after being declared cured was found to be Sputum Smear or Culture positive [8]. All these investigations were performed at the TB Laboratory accredited by Central TB Division, Government of India.

### Data collection, management and statistical analysis

Random Allocation Sequence was generated by a Senior Research Fellow, who also kept secure the codes of the intervention and was not involved in the distribution of the packets and none of the participants, care providers, clinical and laboratory investigators (except for the Senior Research Fellow) were informed of the treatment assignment. Packets containing the two interventions were prepared in advance according to the randomization numbers, arranged in serial order and distributed consecutively to the patients according to their entry into the trial.

As per Revised National Tuberculosis Control Program annual report (2013), 12% of PTB patients receiving category-I ATT had relapse. Assuming that vitamin D supplementation for 24 months (6 months with ATT and 18 months post ATT), would reduce the relapse by 6% by the end of two years from diagnosis. To detect a 6% reduction (12% vs 6%) in the relapse between the two arms in a two-sided test with 5% alpha error and 80% power, 356 participants were required in each group (as per the nQuery Advisor Version 2.0). Giving an allowance of 5% each for losses in follow-up and MDR detection after randomization, 396 participants would be required per group. Therefore, we need to randomize about 800 participants.

The data was analyzed using STATA version 16.0.0. Blood, urine and radiological parameters were analyzed using parametric and non-parametric analysis. Parametric data was analyzed using Student’s t-test, while non-parametric data was analyzed using Wilcoxon rank sum test. Sputum smear and culture data was analyzed using Log rank test as all covariates were balanced in the two groups at baseline. for equality of survivor functions. Relapse data was analyzed using Log-rank test for equality of survivor functions in STATA. Cox regression was used to calculate the hazard ratio and 95% confidence interval for the time to event data.

The protocol was followed as per the Good Clinical Practice standards and Institutional Ethical Guidelines. The protocol was approved by Institutional ethics committee of all three centers (Reference number: AIIMS IEC-256/06.05.2016, RP-1/2016). Written informed consent was taken from all the participants. For participants below the age of 18, written informed consent was provided by their legal guardians. The trial protocol was registered with the Clinical Trial Registry–India. Participants were screened and recruited from February 1, 2017 and followed up till February 27, 2021.

## Results

**Fig 1:**
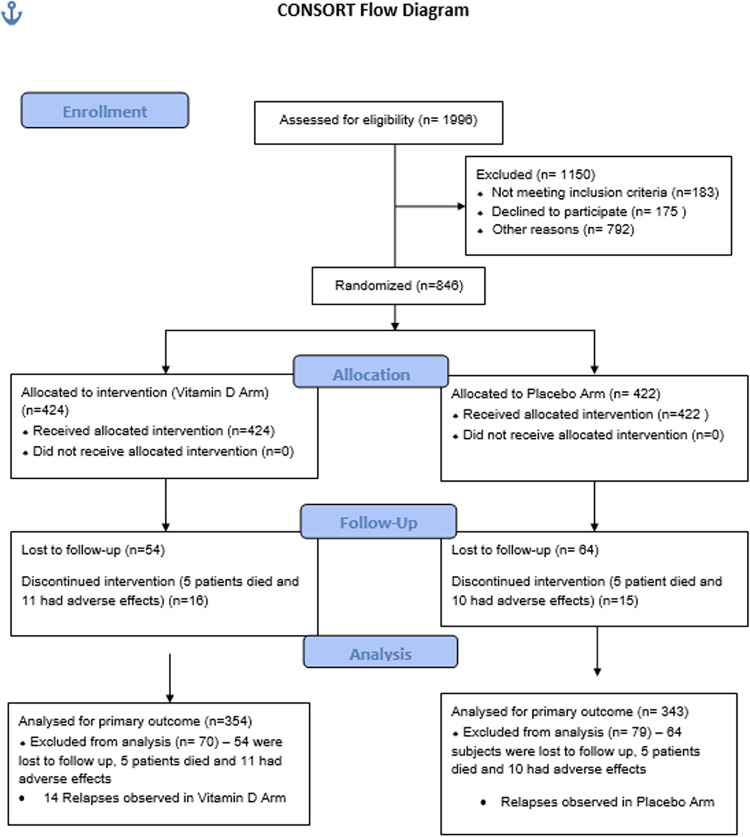
Flow chart showing trial profile. Shows the Trial Profile of the study.

A total of 1996 participants were screened and recruited from February 1, 2017 and followed up till February 27, 2021 at three tertiary care hospitals in India. Of these 1150 participants were excluded and 846 were randomly assigned to the vitamin D group (n = 424) and placebo group (n = 422) (Fig 1).

### Demographic profile

Both the groups saw a preponderance of Male participants (65% & 66%;). The mean age was also comparable (29.2 Vs 29.4; p:08).

Baseline and demographic characteristics did not demonstrate any significant difference between the two groups (Table 1). Baseline BMI is shown in Table 1, with an overall mean of 19.3±3.7 kg/m2. The baseline BMI amongst the two groups did not display any statistically significant difference (p-0.95). A majority of the participants in both groups were deficient in Vitamin D (p). 25(OH)Vitamin D levels amongst the two groups also did not reveal any statistically significant difference (p = 0.156). Similar trends were observed for calcium levels (p = 0.951), phosphate (p = 0.335), urea (p = 0.175), creatinine (p = 0.160), iPTH (p = 0.377) and hemoglobin (p = 0.957).

**Table 1:** Baseline and demographic characteristics.

Demographic Profile (n = 846) (MEAN ± SD)							
Observations	Vitamin D arm (n = 424)		Placebo arm (n = 422)		p-value	Overall (n-846)	
Gender	Male– 276(65%)	Female– 148 (35%)	Male– 279 (66%)	Female– 143 (34%)		Male– 555 (65%)	Female– 291 (35%)
**Age (in years)**	29.2 ± 11.6		29.4 ±11.4		0.821	29.3 ± 11.5	
**Weight (Kg)**	48.7 ± 9.5		49.1 ± 9.8		0.553	48.9 ± 9.6	
**Height (cm)**	158.3 ± 11.1		158.9 ± 10.8		0.457	158.6 ± 10.9	
**BMI (Kg/m** ^**2**^ **)**	19.3 ± 3.9		19.3 ± 3.6		0.955	19.3 ± 3.7	
**Calcium (mg/dl)**	8.8 ± 0.6		8.7 ± 0.6		0.951	8.8 ± 0.7	
**Phosphate(mg/dl)**	3.5 ± 1.0		3.4 ± 0.9		0.335	3.4±1.0	
**25OH Vitamin D**_**3**_ **(ng/ml)**	(3–63.5) 12.4		(2.6–61.1) 11.6		0.156	14.2 ± 8.7	
**iPTH**	(1–229.3) 17.8		(4–115.8) 15.4		0.377	21.3±16.3	
**Hemoglobin (gm/dl)**	11.9 ± 1.9		11.8 ± 1.8		0.957	11.9 ± 1.9	
**Urea (mg/dl)**	24.3 ± 6.7		24.9 ± 6.5		0.175	24.6±6.6	
**Creatinine (mg/dl)**	0.7 ± 0.3		0.8 ± 0.3		0.160	0.7±0.3	
**Chest X-ray severity**	Normal—8 (%)		Normal—6		0.151	Normal—14	
	Mild– 117		Mild– 144			Mild– 261	
	Moderate- 202		Moderate- 194			Moderate- 396	
	Severe—97		Severe– 78			Severe—175	
**Sputum Smear Profile**	1+ - 212		1+ - 200		0.461	1+ - 412	
	2+ - 92		2+ - 86			2+ - 178	
	3+ - 120		3+ - 136			3+ - 256	

Table 1 shows the Baseline and Demographic characteristics between the Vitamin D and the Placebo group. SD = Standard Deviation

### Clinical profile and clinical status

Analysis of the chest radiographs revealed 46% of individuals having moderately affected lungs with no significant difference amongst the two groups at baseline (p = 0.151). Sputum smear profile at baseline showed 49% of participants had 1+ bacilli (AFB), 21% had 2+ and 30% had 3+, with no significant difference seen among both groups (p = 0.46).

### Primary outcome

#### Relapse

For the final analysis, we took patients who had successfully completed their six months of treatment having been cured of PTB and further completed18 months of follow up (697 out of 846). 149 participants were censored of which 118 were lost to follow-up, 21 had adverse event and 10 patients expired.

**Fig 2:**
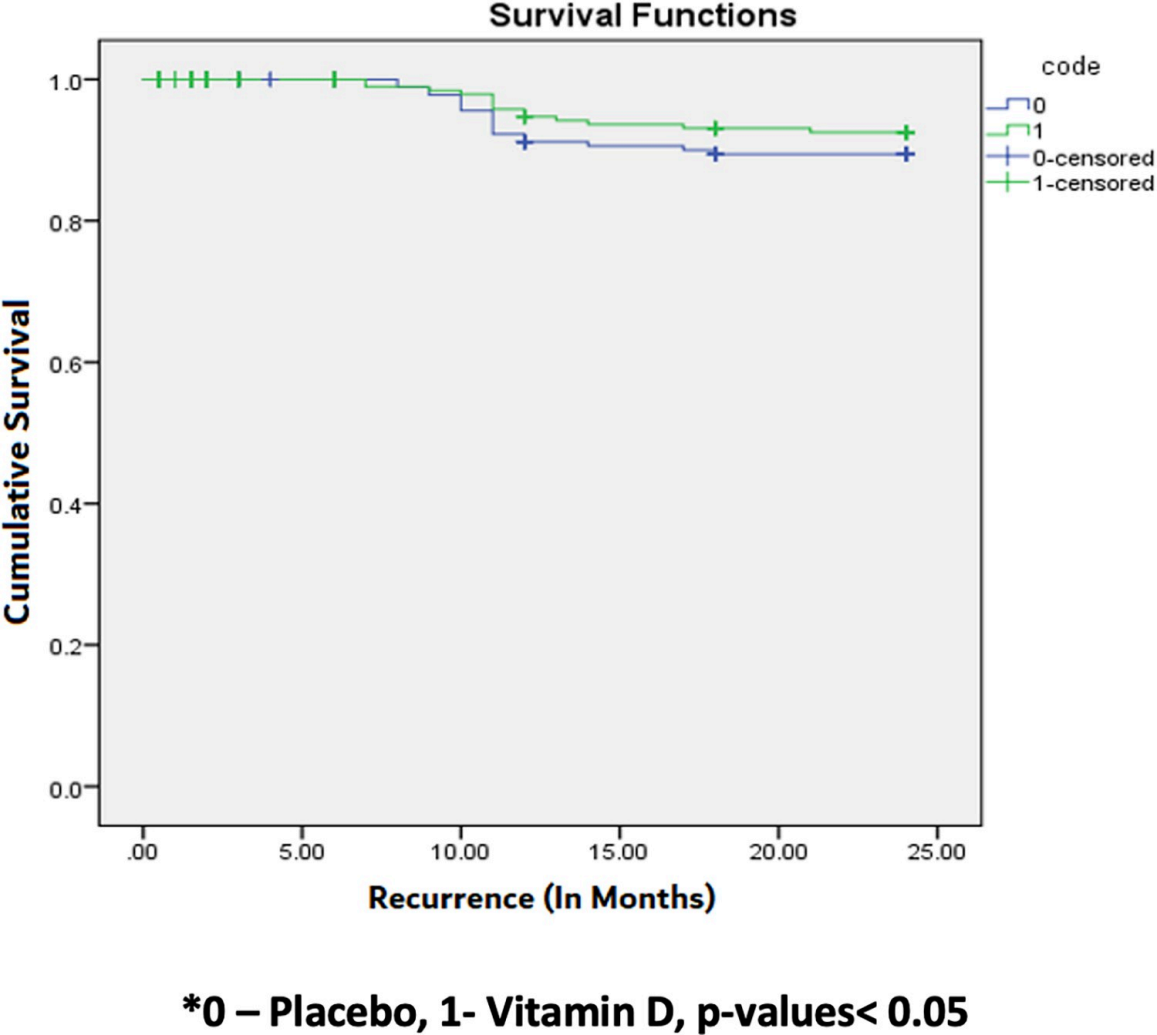
Kaplan Meier graph presenting time to relapse in both the groups. Shows number of relapses in both vitamin D and placebo groups. There are more relapses in the placebo group but the difference is not statistically significant (p-value 0.290).

There were 14 (4%) relapses in vitamin D arm and 19 (5.5%) relapses in placebo arm (n = 697 patients, n = 149 censored), which was however not statistically significant (p = 0.29) (Fig 2). Cox regression revealed a hazard ratio of 0.68 (95% CI: 0.34 to 1.37) having a log rank p-value of 0.29 (Table 2), thus implying a 32% reduction in relapses amongst individuals receiving vitamin D, which is not statistically significant. None of the participants having relapse showed any acquired drug resistance. All the patients with relapse were again initiated on ATT. Most of the individuals having relapse were male (n = 25; 5%) compared to female (n = 8, 3%), which was however not statistically significant (p = 0.25).

**Table 2:** Primary outcome of the study.

No. of participants cured at 6 months	No. of Relapse in Placebo arm (n = 343)	No. of Relapse in Vitamin D arm (n = 354)	Hazard Ratio	95% CI	P- Value
n-697	19 (5.5%)	14 (4%)	0.68	0.34–1.37	0.290

Table 2 shows the total number of relapses observed in the Vitamin D and Placebo group

#### Factors associated with relapse

Further sub group analysis was done to see the relationship between BMI (Asian Indian Criteria) and relapse rate [12]. It was observed that no relapse occurred in the BMI range of ≥25 while 63% (n = 21) of relapse cases were seen in BMI range <18.5, 33% (n = 11) in BMI range 18.5–22.9 and 3% (n = 1) relapse case was seen in BMI range 23–24.9. On Cox regression analysis, hazard ratio was 0.50 (95% CI: 0.2–1.3, p-0.19) which shows more than 50% of relapse cases were observed in <18.5 BMI but results are not statistically significant (p = 0.19).No statistically significant difference was observed in relapse rates amongst the two groups for individuals having higher grade sputum smears (2+ Smear p = 0.21, 3+ Smear p = 0.85) Similarly, no statistically significant difference was observed amongst the two groups for individuals having extensive disease on Chest X-Ray (Moderately Advanced Disease on CXR p = 0.37, Far Advanced Disease on CXR p = 0.96).

### Secondary outcomes

#### Smear and culture conversion

**Fig 3:**
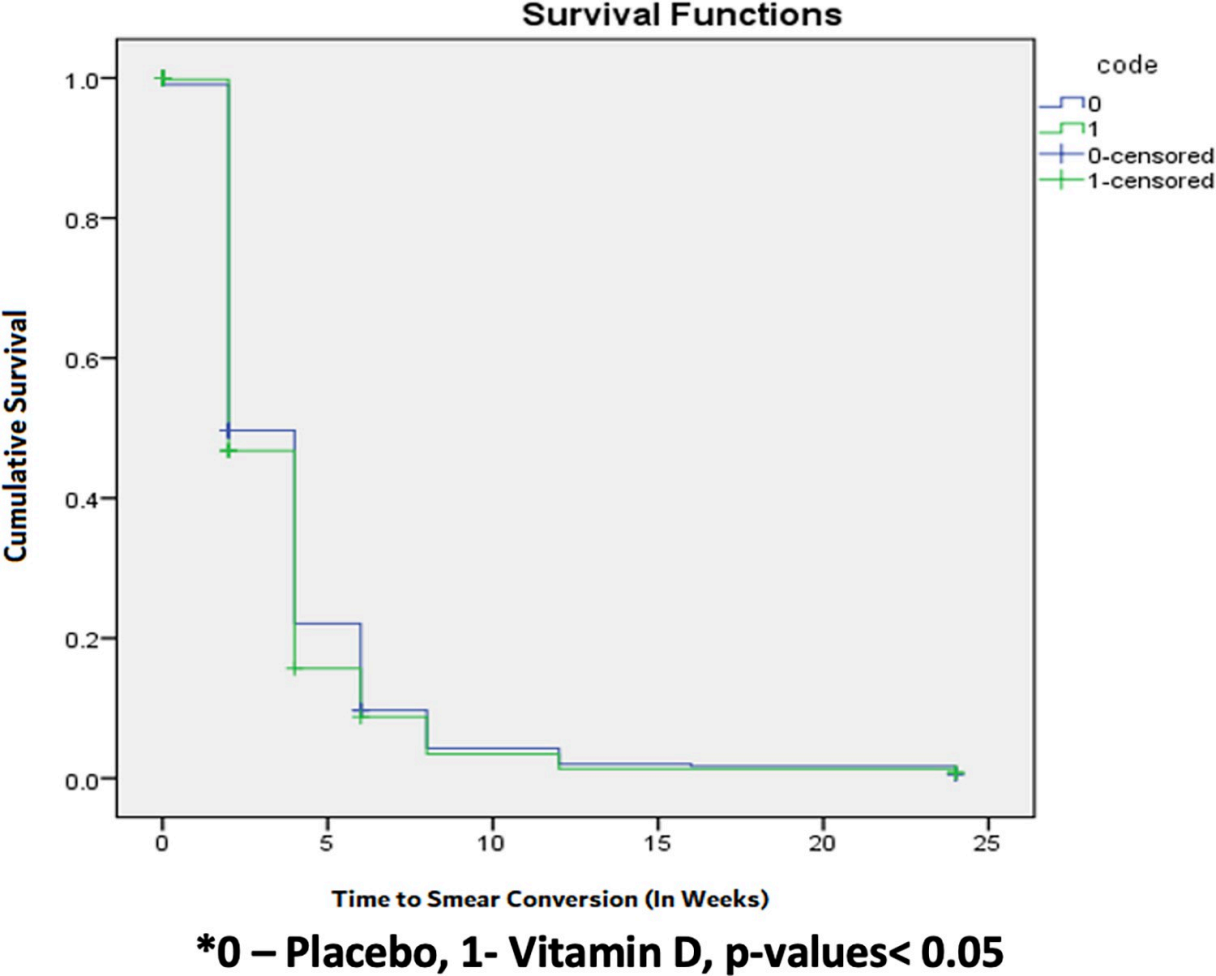
Kaplan Meier graph presenting time to sputum smear conversion in both the groups. Shows time to conversion of sputum smear in both vitamin D and placebo groups. There is no significant difference in the time to sputum smear conversion between the two groups (p = 0.358).

**Fig 4:**
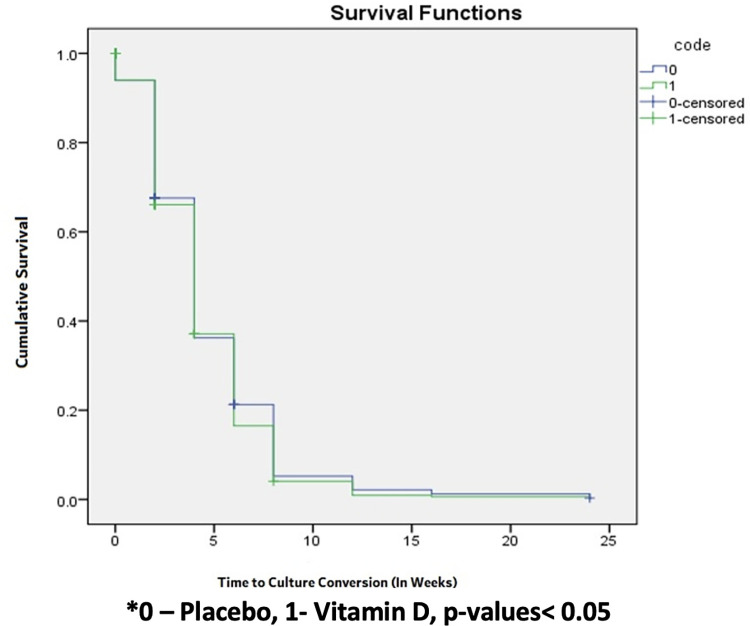
Kaplan Meier graph presenting time to culture conversion in both the groups. Shows Time to conversion of sputum culture in both group Vitamin D and Placebo. There is no significant difference in the Time to conversion of (sputum culture) MGIT with p-value is (0.418).

Median time to sputum smear conversion in both groups was two weeks (95% CI 1.7 to 2.2 in vitamin D arm; 95% CI 1.7 to 2.6 in placebo arm) with hazard risk ratio of 1.06 (95% CI 0.92 to 1.23) and log rank p value 0.35 (Table 3, Fig 3). There was no significant difference observed between vitamin D and placebo groups with respect to median time to sputum smear conversion (n = 779 patients, n = 67 censored). Median time to culture conversion for both groups was four weeks (95% CI 3.6 to 4.3 vitamin D arm; 95% CI 3.7 to 4.3 placebo arm) with hazard risk ratio of 1.06 (95% CI 0.91 to 1.24) and log rank p value of 0.42. Again, there was no significant difference seen in the median time to culture conversion (n = 703 patients, n = 143 censored) between the two groups (Table 3, Fig 4).

**Table 3:** Secondary outcomes of the study.

Group (s)	Participant(s)	Median Time to Conversion (In Weeks)	95% CI	Hazard Ratio	95% CI	p-value
**Time to Sputum smear conversion (n-779)**						
Placebo	385	2	1.7–2.6	1.0	0.92–1.23	0.35
Vitamin D	394	2	1.7–2.2	1.06		
**Time to Culture Conversion (n- 703)**						
Placebo	347	4	3.7–4.3	1.0	0.91–1.24	0.418
Vitamin D	356	4	3.6–4.3	1.06		

Table 3 shows the median time to conversion observed between in the Vitamin D and Placebo group

**Fig 5:**
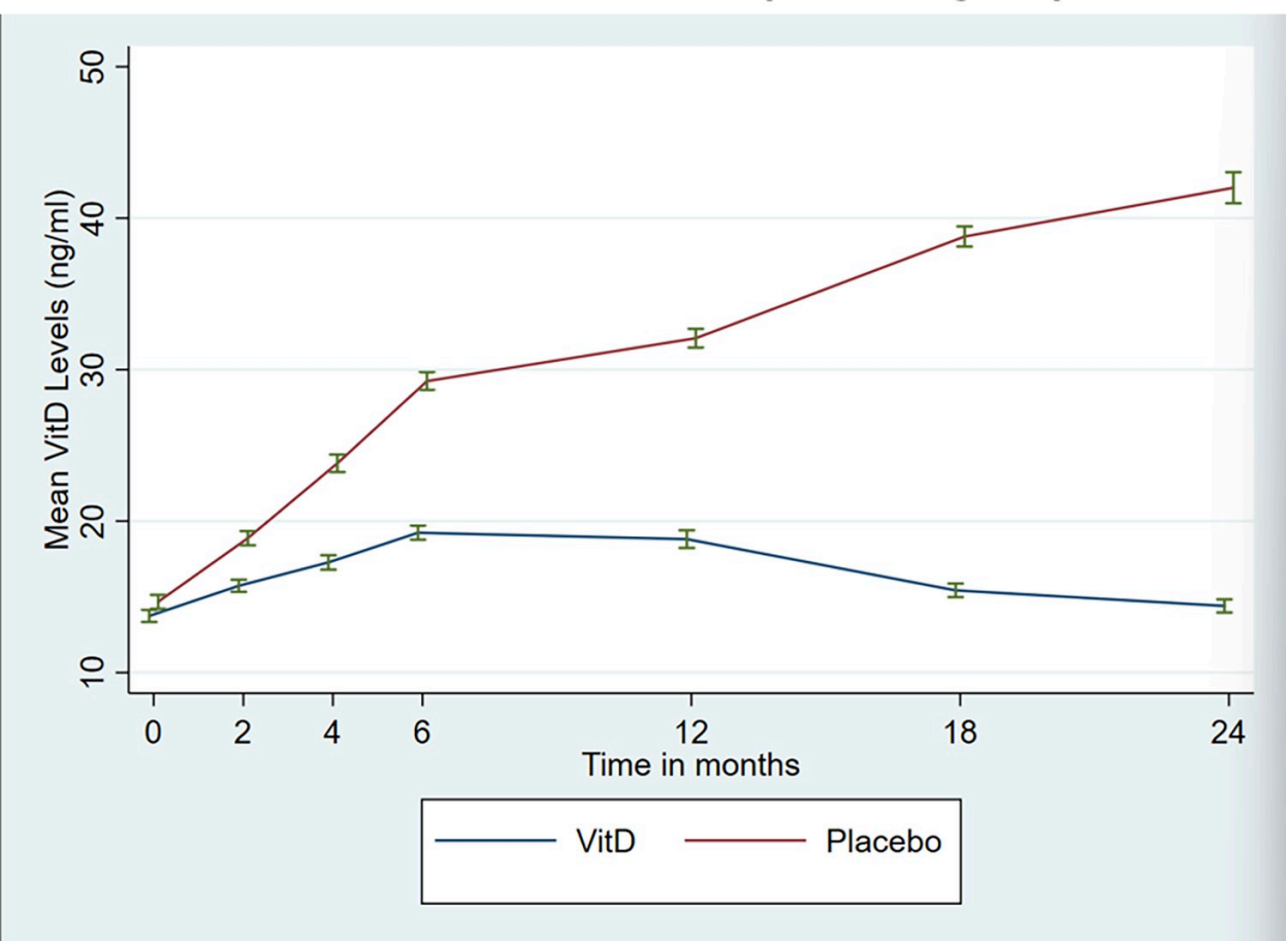
Mean Vit D Levels in both the control and placebo groups. Shows the mean Vitamin D Levels in both Vitamin D and placebo groups. There was significant increase in the Mean Vitamin D levels in the Vitamin D group with time. Error Bars represent 95% C.I.

The intervention arm saw a significant increase in vitamin D levels compared to the placebo arm following supplementation, which was maintained throughout the follow-up at 6^th^, 12^th^, 18^th^ and 24^th^ month (p-value<0.001 at each interval) (Fig 5).

None of the participants in either group developed hypercalcemia. There was no significant difference observed in calcium level during follow up at 6^th^ month (p = 0.62), 12^th^ month (p = 0.52), 18^th^ month (p = 0.17) and 24^th^ month (p = 0.13).

Safety profile (non-serious and serious adverse events) and mortality profile (all-cause deaths) analysis between the two groups (Table 4) showed that 10 (1.1%) patients died during the study [five(1.1%) in the vitamin D group and five (1.1%)in the placebo group]. No death was directly attributable to the study intervention. Excluding death, seven serious adverse events were recorded [4(0.94%) in placebo and 3(0.71) in vitamin D arms). Two (0.4%)patients in vitamin D arm had drug induced liver injury, with 1 patient developing liver abscess, while 4 (0.94%)participants in the placebo arm had serious side effects (one developing ethambutol related ocular toxicity, one developed allergic reaction to the placebo which contained lactose as the active ingredient, one had drug induced liver injury and one developed DRESS syndrome). A total of 14 adverse events were recorded, eight (1.8%) in the vitamin D group and six (1.4%)in the placebo group, all having renal calculi, with none requiring a change in medical therapy (Table 4).

**Table 4:** Adverse events profile.

	Vitamin D (n-424)		Placebo (n-422)			p-value
Variable	No. of Events	Event / 100 person-month	No. of Events	Event / 100 person-month	Hazard Ratio (Vitamin D vs Placebo) 95% CI	
**Death**	5	0.10	5	0.11	0.96 (0.2–3.3)	0.95
**Serious Adverse Event**	3	0.06	4	0.06	0.96 (0.1–4.7)	0.96
**Adverse Event (Renal Calculus)**	8	0.17	6	0.13	1.3 (0.4–3.7)	0.61
**Hypercalcemia**	0	0	0	0	NC	-
**Hypervitaminosis D**	0	0	0	0	NC	-

Table 4 shows the adverse effect profile observed in the Vitamin D and Placebo group

*NC indicates that the risk ratio could not be calculated due to no events in both the groups

## Discussion

At the initiation of our study, we observed most of our patients suffering from active PTB being deficient in vitamin D with mean value at 14.2 ± 8.7ng/ml (Normal:25–80 ng/ml). This is in agreement with similar studies across the globe; a study in US adults acknowledged the inverse relationship of vitamin D levels with the risk of latent TB infection (LTBI) and another in Sudan connected low vitamin D Levels with increased risk of PTB [13, 14]. In view of its anti-inflammatory and anti-microbial qualities as elucidated by Coussens et al, one can make the assumption for Vitamin D deficiency to be a risk factor for the development of PTB [15]. However, a large study in Mongolia involving 8851 children revealed no beneficial effect of Vitamin D supplementation in limiting the risk of TB infection despite the Vitamin D levels reaching physiologic levels [16]. In our study, supplementation of high-dose oral vitamin D was safe and led to a substantial increase in plasma vitamin D concentrations in the intervention arm, but did not contribute significantly towards prevention of relapse. Although the number of relapses in the group without vitamin D supplementation was less, the difference was not statistically significant. Similar findings were seen in a meta-analysis of 8 trials, where despite a significant increase in vitamin D levels, no beneficial effect of vitamin D as adjunctive treatment for TB was observed. [17] We observed relapses in our study to be more common in male participants (n = 25, 5%) compared to female participants (n = 8, 3%), which however was not statistically significant. Individuals having BMI >25 in both the groups were observed to have lower risk of relapse, a phenomenon which is also commonly seen in our clinical practice. One possible explanation for such a phenomenon may be due to the differential biophysical stresses in the lungs of these individuals, wherein young males having lower BMI are more predisposed to enlarging apical bullae, which are easy targets for TB reactivation. Studies in this purview also suggest the same. [18] Another explanation for the same might be the phenomenon of malabsorption, as a result of which there is inadequate absorption of the sufficient amounts of anti-tubercular drugs from the small intestine. These individuals, having being exposed to inadequate amount of anti-tubercular therapy, are rendered more susceptible to future relapses [19].

With regards to the therapeutic value of vitamin D in the treatment of TB, increase in vitamin D levels in serum as a result of supplementation did not translate to significant improvement in time to sputum smear and culture conversion in comparison with the placebo group. This is in agreement with similar studies investigating the relationship between vitamin D and PTB [7]. Wejse et al, did not observe any beneficial outcome of vitamin D supplementation on TB score, weight gain, sputum conversion, or mortality [20]. A study in Georgia confirmed the inverse relationship between vitamin D levels and risk of TB, but failed to show enhanced sputum Mycobacterial clearance in patients receiving high dose vitamin D [21]. According to Martineau et al, vitamin D did not improve time to sputum conversion in the study population, however, sputum conversion was significantly faster in patients with the tt genotype of the TaqI polymorphism of the Vitamin D Receptor gene [6], a finding which was later confirmed by the meta-analysis performed by Zhang et al. [22] In contrast, Tukvadze et al, could not confirm whether the allele resulted in a faster sputum culture conversion for patients on vitamin D treatment. However, their study was significantly underpowered [6, 21]. Certain peers attributed the negative results in the studies of Martineau et al, and Wejse et al, to the low dosage of the intervention [6, 20]. In order to address this limitation, as well as keeping in mind the higher prevalence of vitamin D deficiency in our country as compared to the western world, we supplemented our intervention group with high dose of Vitamin D (60,000 IU), but even this change did not translate to a faster sputum mycobacterium clearance [23]. Of interest is the meta-analysis of RCT of adjunctive vitamin D in patients with Pulmonary TB which demonstrated that although supplementation did not influence time to sputum culture conversion, but did modestly accelerate sputum smear conversion, along with accelerated sputum culture conversion of individuals having MDR-TB [24]. One has also to take into consideration the interesting trend showing failure of Vitamin D supplementation on a myriad of clinical disorders to bring about a beneficial effect, including in COVID-19 infections [25]. In light of all such studies, it becomes evident that additional factors are involved in bringing about the supportive role of Vitamin D to innate anti-microbial immune response. Further studies with sufficient sample size are required in this purview, which might include the role of other nutrients in complementing Vitamin D in preventing TB relapse, as well as the role of genetic polymorphisms in genes involved in the Vitamin D metabolism and Vitamin D Receptor polymorphism in bringing about a differential therapeutic effect in such subjects [26, 27].

No significant difference was detected in the safety profile (non-serious and serious adverse events) and mortality profile (all-cause deaths) between the two groups. The most reported non-serious adverse event in 14 patients out 846 was renal calculi. Adverse events and deaths were evenly distributed between the two groups.

Our study has several strengths, including a large sample size (n = 846) with an appropriate period of follow up (2 years). Participants also received a substantial dose of vitamin D to avoid the possibility of false negative results as criticized by some in the earlier mentioned studies. The participants were closely followed up regularly throughout the study period and the administration of the intervention was directly observed, ensuring good compliance to medications and thus eliminating the confounding effect of non-compliance contributing to relapse. The proportion of missing outcome data was small and comparable in both the groups. Therefore, our findings have a high degree of validity.

The main limitation of our study was that sputum cultures for tuberculosis was not done after 6 months of routine follow up in all patients, which can have led us to miss a few cases of relapse. Sputum samples were examined for Acid Fast Bacilli, which has a relatively lower sensitivity compared to investigations such as GeneXpert, which could have further added up to the limitations [28]. Also, the sample size was calculated with the expectation of a substantially higher relapse rate in the control arm. This, along with the fact that 17% of the initially recruited patients could not complete the trial, with the COVID pandemic being responsible in large part, may have rendered our study potentially underpowered.

## Conclusions

Vitamin D supplementation showed no benefit over placebo in preventing relapse of PTB and in reducing time to sputum smear and culture conversion. There were no major adverse reactions observed in either the vitamin D or placebo arm which could be ascribed to the intervention.

## Supporting information

S1 Checklist CONSORT 2010 checklist of information to include when reporting a randomised trial*. (DOC)

S1 File Research protocol. (DOCX)

## List of abbreviations

AIIMS: All India Institute of Medical Sciences
PTB: Pulmonary Tuberculosis
TB: Tuberculosis
ATT: Anti-tubercular treatment
DOTS: Directly Observed Treatment, Short Course
RNTCP: Revised National Tuberculosis Control Program
ICMR: Indian Council of Medical Research
DHR: Department of Health Research
BMI: Body Mass Index
