# Supplementary material for: Prevention of relapse in drug sensitive pulmonary tuberculosis patients with and without vitamin D3 supplementation: A double blinded randomized control clinical trial
Source: PLoS One. 2023 Mar 30;18(3):e0272682. doi: 10.1371/journal.pone.0272682 (PMC10062618; doi:10.1371/journal.pone.0272682)
Supplement: S1 File — (DOCX) [file pone.0272682.s002.docx]

**RESEARCH PROTOCOL**

***Part 1***

**Project summary:**

Pulmonary Tuberculosis (PTB) and Vitamin D deficiency (VDD) are national problems. Studies on vitamin D as an adjuvant to anti tuberculosis treatment (ATT) are important due to historical use and benefits to the community in case this cost effective help contain mycobacterium (*Mtb)* infection. As most of Indians are suffering from either vitamin D insufficiency or deficiency so, usual dose is, 60,000 IU of cholecalciferol weekly for 1st two months then every 15 days for the 4 months and then 60,000 IU every month for 18 months. The Calcium carbonate supplementation will also be given.

Serum 25(OH) Vitamin D, parathormone, S. Calcium, Phosphorus, Alkaline Phosphate, LFT and Urine Calcium (spot) will be measured at baseline, 8^th^ and 24^th^, 48^th^  & then every 24^th^ week. USG abdomen will be done at baseline, 6 months and 24 months to detect renal stones. Sputum smear for AFB, MTB culture & drug sensitivity to find out MDR TB & will be done as per RNTCP (Revised National Tuberculosis Control Programme) guidelines. The CAT I ATT will be given as per RNTCP guidelines. The aims of study are to study the effect of Vitamin D3 supplementation in the prevention of relapse in *Category* I (CAT I) treated Pulmonary Tuberculosis patients & study the cost effectiveness of Vitamin D3 supplementation. The study design would be a randomized double blind controlled clinical trial. In this study during first 6 months in one group we will receive ATT plus Vitamin D3 supplementation and other group will receive ATT with placebo. The next 18 months we will follow up these patients for 18 mounts after ATT & one group we will receive Vitamin D3 supplementation and other group will receive placebo. The sample size for this study will be about 800 patients.

**General Information:**

1. **Title:** Prevention of relapse in Drug Sensitive Pulmonary Tuberculosis patients with & without Vitamin D3 supplementation: A randomized double blinded control clinical trial.
2. **ID number: CTRI/2021/02/030977 (** date of first registration-03/02/2021**).**
3. **Date:**
4. **Name and address of sponsor / funder:**  Department of Health Research, Ministry of Health and Family Welfare, Government of India.
5. **Name and address of investigators / research sites / clinical laboratories:**

Sanjeev Sinha^1*^, Himanshu Thukral^1^, Imtiyaz Shareef^1^, Devashish Desai^1^, Binit Singh^1^, Bimal Kumar Das^2^, Sahajal Dhooria^3^, Rohit Sarin^4^, Rupak Singla^4^, Saroj Kumari Meena^4^, R.M. Pandey^5^, Shivam Pandey^5^, Sunil Sethi^6^, Ashumeet Kajal^3^, Rakesh Yadav^6^, Ashutosh Nath Aggarwal^3^, Sanjay Bhadada^3^, Digambar Behera^3^

**Authors Details:**

^1^Department of Medicine, All India Institute of Medical Sciences, Ansari Nagar, New Delhi 110029,

^2^Deparment of Microbiology, All India Institute of Medical Sciences, Ansari Nagar, New Delhi 110029,

^3^Department of Pulmonary Medicine, Post Graduate Institute of Medical Education and Research, Chandigarh, India,

^4^Department of Respiratory Medicine, National Institute of Tuberculosis and Respiratory Diseases, New Delhi, India,

^5^Department of Biostatistics, All India Institute of Medical Sciences, Ansari Nagar, New Delhi, India

^6^Department of Medical Microbiology, Post Graduate Institute of Medical Education and Research, Chandigarh, India

***Corresponding author:**

Dr. Sanjeev Sinha, MD

Department of Medicine,

All India Institute of Medical Sciences,

New Delhi, India.

Email ID: drsanjeevsinha@gmail.com

Contact Phone No.: +919810164416

**Introduction:**

Tuberculosis and vitamin D deficiency (VDD) are important public health problems in our country (1,2,3) In recently published studies from Indian centres, a remarkably high proportion of apparently healthy subjects especially from urban Indian cities have been classified either as vitamin D insufficient or deficient by using serum 25(OH) Vitamin D cut off levels of < 32 ng/ml and < 20 ng/ml respectively(4,5). Presence of VDD has been described in all age groups ranging from infancy to elderly in Indian population (6,7,8). The deficiency is also observed in rural area albeit with lesser severity (9) The functional significance of the widespread VDD deficiency in Indians is under investigation. Recently, interesting data have been generated on the potential implication of VDD in Indians on skeletal system as osteoporosis (6,7) and osteomalacia (10) and intestinal calcium absorption(11). Worldwide, there has been interest in the extra skeletal effects of VDD and its role has been implicated in disorders such as autoimmune type 1 DM, carcinoma of the breast/prostate, susceptibility to bacterial/mycobacterium infections and musculoskeletal strength (12). However, there is paucity of systematic data on these extra skeletal aspects of VDD based on strong evidences supported by double blind, placebo control randomized trials (RCT). Recently, our centre has reported effect of cholecalciferol supplementation on musculoskeletal strength in apparently healthy Indian females with VDD (11,5)

With wide awareness of VDD in general populations physicians have been increasing providing cholecalciferol supplementation to apparently healthy asymptomatic individuals with biochemical 25(OH) Vitamin D deficiency. The vitamin D is supplemented in various doses with the aim to maintain serum 25(OH) Vitamin D between 20-32 ng/ml. One of the common regimens used is oral cholecalciferol 60,000 IU/week for eight weeks followed by supplementation with 60,000 IU fortnightly along with calcium carbonate on daily basis.

The historical evidences of TB dates back to 7000 BC in the skeletal remains of Neolithic age human, Egyptian mummies (3000 BC)(13). Robert Koch used a new staining method and discovered fungus like bacteria in the sputum of patients' with pulmonary TB (PTB), named it ‘tubercle bacillus’ and received Nobel Prize for it. Subsequently it was named as ‘Mycobacterium tuberculosis’ (*Mtb*) due to its mycolic acid content in the cell wall (‘myco’ means “waxy” in Latin). Following the discovery of X-ray in 1895 by Roentgen, chest radiography was used to diagnose PTB (14). True to the perception there has been remarkable reduction in the incidence of TB and related mortality (15). However, the disease still accounts for the major morbidity and mortality in the world with 1.3 millions TB related deaths reported in 2012 (15).

**Prevalence of tuberculosis: Global and Indian Scenario Global**

India, China, South Africa, Indonesia and Pakistan are the major countries affected with the disease (15). It was estimated that approximately one third of the global population was infected with mycobacterium tuberculosis and 5-10% of them would have developed clinical disease during their life-time. In 2010-2011, there were 12 million cases of TB in the world comprising of 5.7 million new cases and 2.7 million sputum positive cases (15). The disease affected 2.9 and 0.5 million women and children respectively. Asia (58%) and Africa (27%) have the highest burden of TB and India and China jointly account for 38% of the global TB burden (15). Of the estimated 52.8 million deaths globally in 2010, tuberculosis contributed in 1.2 million. Thus, even after a century of discovery of *Mtb*, TB remains a major cause of mortality in the world (15). In view of this situation, the World Health Organization (WHO) declared TB as a global emergency in 1993 and realizing the huge burden of the disease, several world agencies are contributing towards the cause of the disease. WHO in 1993, devised and recommended Directly Observed Treatment Short course chemotherapy (DOTS) for the management of the disease. Briefly, the DOTS comprised of diagnosis of TB by sputum smear, uninterrupted supply of high quality anti-TB drugs, its supervised intake and accurate reporting/recording of the cases. Recently, multi drug-resistant TB (MDR-TB) has been recognized as a major public health problem. There is no systematic data on the prevalence of MDR tuberculosis in various populations in view of lack of easy access to facilities for Drug sensitivity testing. In 2012, globally 3.6% of newly diagnosed and 20% of previously treated TB cases had MDR-TB (Global tuberculosis report 2013, WHO). However, in eastern Europe and central Asia more than 20% of new and more than 50% of previously treated patients with TB may have MDR-TB (15).

**Rationale and background information:**

Prevention of relapse in Category I (Drug Sensitive) treated Pulmonary Tuberculosis patients by using Vitamin D has not been studied so far in India. From all the available literature it can be conclude that, serum level of 25(OH) D is the most important for the effects of vitamin D either for infectious disease (PTB) or for other disease related to bone health. The aims of study are to study the effect of Vitamin D3 supplementation in the prevention of relapse in *Category* I (CAT I) treated Pulmonary Tuberculosis patients. We will follow up all PTB patients 18 months after giving six months of ATT.

**Study goals and objectives:**

1. To study the effect of Vitamin D3 supplementation in the prevention of relapse in *Category* I (CAT I/ Drug Sensitive) treated Pulmonary Tuberculosis patients.

**Study design:**

The study design would be a randomized, double blind, clinical trial. In this study during first 6 months, participants in the study group suffering from PTB will receive ATT plus Vitamin D3 supplementation and the control group will receive ATT with placebo. Both will be followed up for for 18 mounts after completion of ATT.

In one group, Vitamin D3 supplementation would be given for 18 months & in other group, placebo will be administered for 18 months after ATT.

Inclusion criteria:

- Age ≥15 years
- Diagnosed PTB case as per RNTCP guidelines
- Able to give informed written consent
- Willing to participate in the study
- No major medical or surgical illness

Exclusion criteria:

- Not willing to participate
- Age > 60 years
- With major medical or surgical illness (see below)
- Suffering from MDR-TB
- Human Immunodeficiency Virus (HIV) infection,
- BMI < 15kg/m2,
- Diabetes mellitus,
- Chronic alcoholism,
- Pregnant and lactating women,
- Receiving any kind of vitamin D / calcium supplementation
- Having any disorder of bone mineral homeostasis,
- Those refusing consent.

**Methodology:**

All newly diagnosed sputum AFB smear positive patients with Cat-I PTB, aged between 18-60 years will be included. Patients with alcoholism as per CAGE criteria, pregnancy, lactation, on vitamin D and calcium supplementation or on any other drug affecting bone mineral homeostasis during past six months, any other clinically overt disease, body mass index (BMI) < 15.0 kg/m2, seriously ill requiring hospitalization and outstation patients unlikely to come for regular follow up were excluded. All the patients will be called at the tertiary centre and will undergo the following.

**Pre-randomization Screening:** Pre-randomization investigations included assessment of hepatic and renal function, serology for human immunodeficiency virus (HIV), hepatitis B (HBV) and C (HCV) and fasting plasma glucose. Repeat assessment of AFB smear positivity will be done in the early morning sputum sample brought by the patient and again on-spot in sterile container. Xray chest, electrocardiogram, complete hemogram and urine microscopy will also done. Patients with HIV, HBV and HCV antibodies, serum creatinine > 1.5 mg/dl, total serum bilirubin > 1.5 mg/dl and aspartate aminotransferase (AST), alanine aminotransferase (ALT) > one and half times the normal values and those with impaired fasting glucose and diabetes as per WHO criteria (plasma glucose > 109 mg/dl and > 125 mg/dl respectively) will be excluded. All screening was completed within three working days.

**Randomization, concealment, blinding and supplementation:** Subjects will be assigned to two intervention groups by block randomization with a block size of four using computer program (http:www.graphpad.com/quickcales/random2.cfm). Packets containing the two interventions will be prepared in advance according to the randomization numbers, arranged in serial order and distributed consecutively to the patients according to their entry into the trial. The codes of intervention in the packets will be kept with an investigator not involved in distribution of the packets. Patients, caregivers and laboratory staff will be blinded to the intervention assigned to the patients and their baseline and follow up serum vitamin D and intact parathormone (iPTH) values until the completion of the last follow up. Subjects in the active intervention arm will be received a cholecalciferol sachet orally (60, 000 IU D3/week for first 8 weeks along with two tablets of calcium carbonate given daily for 6 months. Each tablet of calcium carbonate contained 500 mg of elemental calcium and 250 IU D3. Patients in the control group will receive double placebo of sachets and tablets for the corresponding duration. The placebos for the calcium and cholecalciferol will be made of lactose. Active and passive interventions will be matched in terms of their physical appearance and taste. Packets containing interventions will be provided at weekly interval for the initial 8 weeks and then every fortnightly for the next 8 weeks, followed by monthly intervals thereafter till the 24^th^ month. Weekly intake of cholecalciferol/placebo sachet will be given under supervision of the physician with a glass of milk during first eight weeks and patients will be advised to take prescribed calcium/placebo tablet with meals, in the morning and evening. The cholecalciferol supplementation schedule used will based on our previous experience with vitamin D supplementation in healthy north Indians which resulted in optimal serum 25(OH) Vitamin D levels at 8 wks. The Institutional Ethics Committee approval will be taken for the study protocol and written informed consent will be obtained from all the study subjects enrolled in the trial.

**Baseline investigations:** All the patients will be assessed for clinical features including cough, haemoptysis, and severity of changes on X-ray chest as per the American Thoracic Society (ATS) criteria (American Thoracic Society, 1961) Sputum AFB culture and anti-tubercular drug sensitivity will be performed for all subjects. Blood will draw in the fasting state between 0800 and 0930 h and centrifuged under cold conditions at 3000 RPM for 15 min. Serum will be stored in multiple aliquots at -20C for measurement of serum total calcium, inorganic phosphorus, alkaline phosphatase (SAP), albumin, 25(OH) Vitamin D and iPTH in batches. Biochemical osteomalacia was defined as SAP > 240 IU, 25(OH) Vitamin D <20.0 ng/ml and iPTH > 65.0 ng/ml.

**Anti-tubercular treatment:** Participants in both the intervention arms will receive standard care from local DOT centre. Medical officer of each DOT centre prescribed supervised therapy free of cost during intensive and continuation phase as per the RNTCP guidelines. Briefly, therapy involved thrice weekly isoniazid, rifampicin, ethambutol and pyrazinamide during intensive phase in the first two months and thrice weekly INH plus rifampicin during continuation phase for next four months (2H3R3Z3E3 + 4R3H3). Those with persistent sputum AFB smear positive at two months of therapy will be provided an extra month of intensive therapy

**Follow up including safety considerations:**

Patients will be called for follow up at weekly intervals for eight weeks and thereafter at four weekly till 24 weeks for morning sputum AFB smear and culture on each visit. To assess relapse, patients will be followed up after completion of ATT therapy at six monthly intervals for additional 18 months (total follow up of 24 months from start of ATT therapy) with sputum AFB smear and *Mtb* culture will performed on each visit. All the biochemical investigations including serum 25(OH) Vitamin D and iPTH will be estimated 8th, 24th and 48th weeks following initiation of intervention. However, serum total calcium, phosphorus, albumin and alkaline phosphatase, liver function tests will be repeated every two weeks interval during intensive phase and thereafter at 4 weeks interval during continuation phase of ATT. Urine Ca/Cr ratio was assessed at 4 wk intervals till 24^th^ week of intervention.

**Cholecalciferol and calcium compliance and safety:** Subjects will be called every week during first eight weeks of cholecalciferol and calcium supplementation and instructed to bring the leftover tablets that will not consumed. The compliance of the tablet will be assessed using a pill-counting method. A medical person, supervised the intake of sachet with a glass of milk on each visit. Compliance will also be assessed retrospectively after the randomization code will be broken by their serum 25(OH)Vit-D levels in the blood samples drawn at 8 weeks of enrolment. To assess the safety of the supplementation, serum total calcium, phosphorus and SAP, will be measured every fortnight during intensive phase and thereafter at every 4 wks till 24th wks of intervention. To assess the possibility of hypercalciuria, urine calcium/creatinine ratio will be measured in the first sample in the morning at every 4 wks. Hypercalcemia will be defined as serum total calcium (adjusted for albumin) over 10.6 mg/dl (Gallagher *et al.,* 2012). Severe hypercalcemia will be defined as serum total calcium > 11.0 mg/dl (Horwitz *et al*.,2010). Urinary calcium/creatinine ratio higher than 0.2 will be defined as hypercalciuria (Sargent *et al*., 1993). Compliance for ATT therapy and the number of subject requiring extended intensive phase therapy will be recorded from the patients’ DOT sheet at the completion of continuation phase of therapy.

**Microbiological assessment**: Sputum AFB smear, *Mtb* culture and assessment of drug

sensitivity will be performed. Briefly, sediment of NALC/NAOH treated sputum sample will be stained for AFB by Ziehl Neelsen method (Laboratory service in TB control. 1998, WHO). Slides will be examined under oil emersion objective and number of AFB observed will be graded from scanty to 3+ as per the recommendation of WHO (Laboratory service in TB control. 1998, WHO). Sputum culture will be carried out using radiometric BACTEC 460 TB detection system (Becton Dickinson, Sparks, MD, USA) till 2010 and subsequently Mycobacteria Growth Indicator Tube (MGIT-960) non-radiometric automated isolation system (Becton Dickinson), and drug resistance will be determined using the Line Probe Assay (LPA) method. Rifampicin and INH sensitivity will be assessed by 1% proportion method and those resistant to both will be considered as MDR (Global tuberculosis report 2012, WHO).

**Biochemical assessment:** Serum total calcium, inorganic phosphorus, and alkaline phosphatase will be measured using standard laboratory procedures on an automated analyzer (Beckman Coulter, Hialeah, FL; Syncron clinical system CX4PRO; normal range: 8.4–10.2 mg/dl, 2.5– 4.6 mg/dl, and 80–240 IU/ml, respectively. Serum 25(OH) Vitamin D will be measured using Radioimmunoassay (DiaSorin, Inc., Stillwater, MN). Serum intact PTH (iPTH) will be

measured using electrochemiluminescence assay (Elecsys-2010; Roche Diagnostics,

Indianapolis, IN); normal range: 15–65 ng/ml. Intra and inter-assay coefficients of

variation for these assays ranged from 3.5–5.0%.

**Deviation from Protocol:**

After 1 year of study initiation, recruited patients were given daily Isoniazid, Rifampicin, Ethambutol and Pyrazinamide during intensive phase in the first two months and daily Isoniazid plus Rifampicin plus Ethambutol during continuation phase for next four months (2HRZE + 4HRE) in accordance with changes introduced in RNTCP during that time.

**Data management and statistical analysis:**

As per Revised National Tuberculosis Control Program annual report (2013), 12% of PTB patients receiving category-I ATT relapsed.  Assuming that vitamin D supplementation for 24 months (6 months with ATT and 18 months post ATT), would reduce the relapse by 6% by the end of two years from diagnosis.  To detect a 6% reduction (12% vs 6%) in the relapse between the two arms in a two-sided test with 5% alpha error and 80% power, 356 participants in each group (as per the nQuery

Advisor Version 2.0). Giving an allowance of 5% each for losses in follow-up and MDR detection after randomization, 396 participants would be required per group. Therefore, we need to randomize about 800 participants.

The data will be analysed using STATA version 16.0.0. Blood, urine and radiological parameters will be analysed using parametric and non-parametric analysis. Parametric data was will be analysed using Student’s t-test, while non-parametric data will be analysed using Wilcoxon rank sum test. Sputum smear and culture data will be analysed using Wilcoxon (Breslow) test for equality of survivor functions. Relapse data will be analysed using Log-rank test for equality of survivor functions in STATA.

**Expected outcomes of the study:**

We expect that Vitamin D supplementation may improve treatment outcomes in patients of pulmonary tuberculosis, specifically in terms of time to sputum smear and culture conversion and reduction in the rate of relapse. Considering pulmonary tuberculosis is a major public health problem in India, such a result would supplement the available armamentarium against tuberculosis and would be of tremendous public health importance.

**Duration of the project:**

4 years

**Novelty of your research project:**

1. Relative importance of research project in a given area:

We suppose that vitamin D supplementation for 24 months (6 months of ATT & 18 months post ATT) would reduce the relapse by 6% at the end of two years from diagnosis of TB. PTB and Vitamin D deficiency are national problems and have priorities of medical research. Studies on vitamin D as an adjuvant to ATT are important due to historical use and benefits to the community in case this cost effective help contain mycobacterium (*Mtb)* infection. From all this literature it can be conclude that, serum level of 25(OH) D is the most important for the effects of vitamin D for infectious disease like TB.

**Applicability of your research project in**

(a) Short term: We suppose that vitamin D supplementation for 24 months (6 months of ATT & 18 months post ATT) would reduce the relapse by 6% at the end of two years from diagnosis of TB.

(b) Long term: Pulmonary tuberculosis (PTB) and vitamin D deficiency are global problems. Studies on vitamin D as an adjuvant to anti tuberculosis treatment are important due to its potential role in containing mycobacterial (*Mtb)* infection. As most Indians are suffering from either vitamin D insufficiency or deficiency so supplementation of vitamin D in PTB patients as an adjuvant to ATT will help in long term to reduce relapse of PTB and decrease overall transmissibility in the population.

**Ethics:**

The protocol will be followed as per the Good Clinical Practice standards and Institutional Ethical Guidelines. The study will commence only after the protocol is approved by DHR, ICMR and institutional ethics committee of all three centers. Written informed consent will be taken from all the subjects. The trial protocol will be registered with the Clinical Trial Registry of India


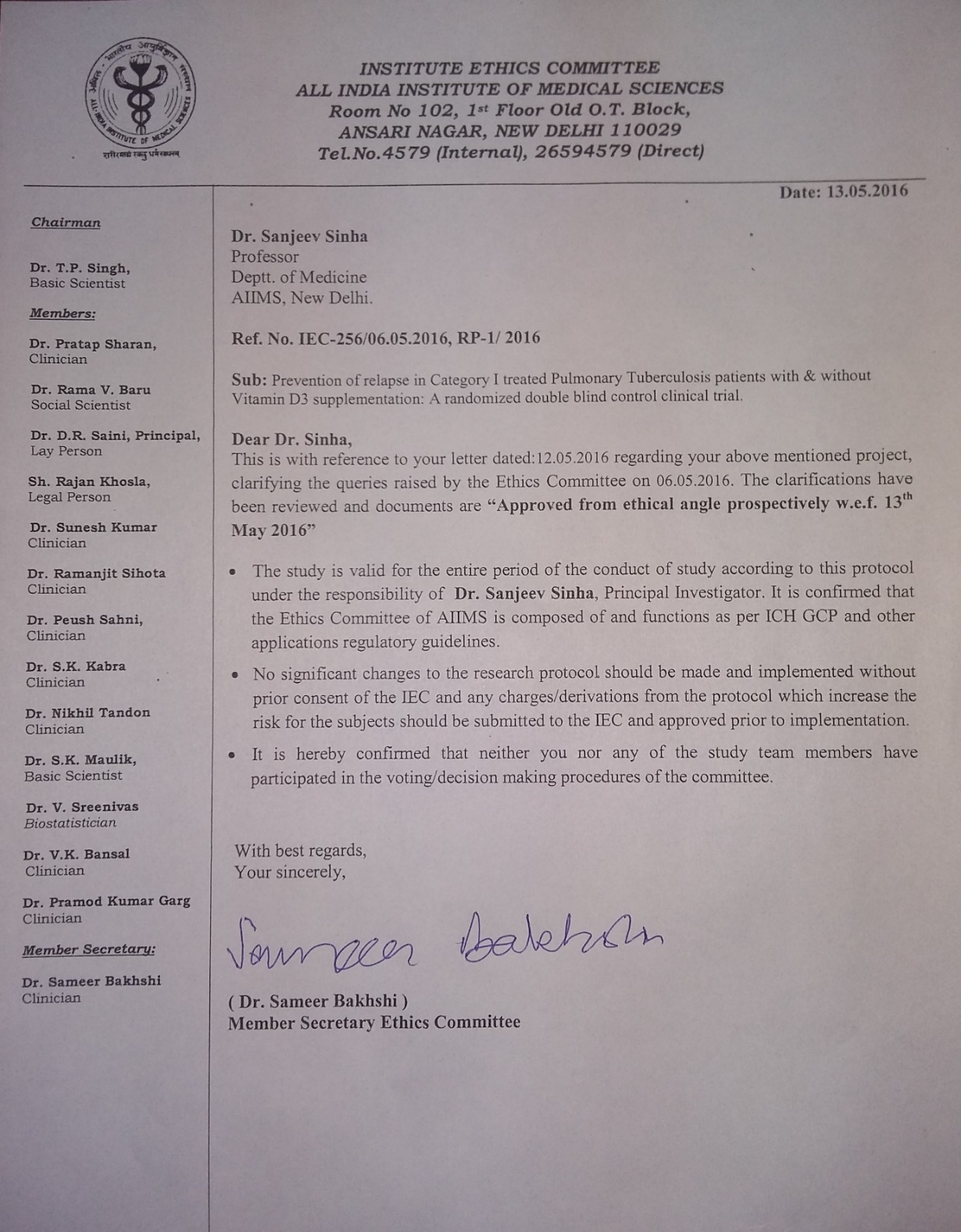


**Informed consent forms:**

*Part 2*

**Budget:**

**Other support for the project:**

**CV of primary investigator:**

**Other research activities of investigator:**

**Financing and insurance:**

The trial protocol was registered with the Clinical Trial Registry – India (CTRI 2021/02/030977 dated February 3, 2021)

**References:**

1. Goswami R,. Mishra S.K & Kochupillai N . prevalence & potential significance of vitamin D deficiency in Asian Indians. Indian J Med Res, 2008a; 127:229-238.
2. Marwaha RK, Tandon N, Reddy DRHK, Aggarwal R, Singh R, Sawhney RC, et al. Prevalence and significance of low 25-hydroxyvitamin D concentrations in healthy subjects in Delhi. Am J Clin Nutr 2005a; 82 : 477-82.
3. Sachan A,Gupta R,Das V,AgarwalA, AwasthiPK, Bhatia V. High prevalence of vitamin D deficiency among pregnant women and their newborns in northern India. Am J Clin Nutr 2005;81:1060-4.
4. Harinarayan CV, Ramalakshmi T, Prasad UV, Sudhakar D, Srinivasarao PV, Sarma KV & Kumar EG . High prevalence of low dietary calcium, high phytate consumption,and vitamin D deficiency in healthy south Indians. Am J Clin Nut, 2007; 85,1062-67.
5. Goswami R, Vatsa M, Sreenivas V, Singh U, Gupta N, Lakshmy R, et al. Skeletal muscle strength in young Asian Indian females after vitamin D and calcium supplementation: a double-blind randomized controlled clinical trial. *J Clin Endocrinol Metab* 2012; 97: 4709-4716.
6. Marwaha RK, Tandon N, Reddy DR, Aggrawal R, Singh R, Sawhney RC, Saluja B, Ganie MA, Singh S. Vitamin D and bone mineral density status of healthy school children in northern India. Am J Clin Nutr 2005b; 82: 477-482.
7. Marwaha RK, Tandon N, Garg MK, Kanwar R, Narang A, Sastry A, Saberwal A, Bandra K. Vitamin D status in healthy Indians aged 50 years and above. J Assoc Physicians India. 2011 ;59:706-9.
8. Harinarayan CV.prevalence of vitamin D deficiency in postmenopausal south India women. Osteoporos Int 2005;16: 397-402.
9. Goswami R, Kochupillai N, Gupta N, Goswami D, Singh S, Dudha A. Presence of 25 (OH) D deficiency in a rural North Indian village despite abundant sunshine. 2008b; 56:755-757.
10. Ray D, Goswami R, Gupta N, Tomar N, Singh N, Sreenivas V. Predisposition to vitamin D deficiency osteomalacia and rickets in females is linked to their 25(OH) Vitamin D and calcium intake rather than vitamin D receptor gene polymorphism. *Clin Endocrinol.,* 2009; 71:334-340.
11. Gupta A, Gupta N, Singh N, Goswami R. Presence of impaired intestinal calcium absorption in chronic hypovitaminosis D and its change after cholecalciferol supplementation: assessment by the calcium load test. J Hum Nutr Diet. 2010;23:54-60.
12. Holick MF, Binkley NC, Bischoff-Ferrari HA, Gordon CM, Hanley DA, Heaney RP, Murad MH, Weaver CM. Guidelines for preventing and treating vitamin D deficiency and insufficiency revisited. *J Clin Endocrinol Metab*. 2012;97:1153-1158.
13. Hershkovitz, I., H. D. Donoghue, et al.. "Detection and molecular characterization of 9,000-year-old Mycobacterium tuberculosis from a Neolithic settlement in the Eastern Mediterranean." PLoS One,2008; 3: e3426.
14. Novelline RA. 1997. *Squire's Fundamentals of Radiology*. Edn 5, Harvard University Press, USA.
15. *Global Tuberculosis Report. Geneva: 2013* World Health Organization (www.who.int/iris/bitstream/10665/75938/1/9789241564502_eng.pdf).
